# The Balint Group Experience for Forensic Mental Health Professionals

**DOI:** 10.1192/bjo.2022.378

**Published:** 2022-06-20

**Authors:** Fraser Arends, Graeme Reed, James FitzGerald

**Affiliations:** 1 Cambridgeshire and Peterborough NHS Foundation Trust, Cambridge, United Kingdom; 2 University of Cambridge, Cambridge, United Kingdom

## Abstract

**Aims:**

Balint groups were initially set up to meet the needs of GPs in better understanding the emotional aspects of complex doctor-patient relationships. They have since been taken up in the training of psychiatrists, GPs, and medical students, having been shown to improve communication skills and sensitise participants to their own psychological processes. Working as a Care Coordinator in a Forensic Community team is a highly challenging role where, by definition, there is the spectre of risk of harm to others. There is very little published data on the use of Balint groups in nursing populations, even less so in the Forensic mental health setting. The aim of this project was to evaluate a longitudinal Balint group for mental health professionals in the Forensic service of Cambridge and Peterborough NHS Foundation Trust, and to report on the perceived benefits to attending in terms of personal and professional development.

**Methods:**

The evaluation used a standardised mixed methods approach, with the sample consisting of members of the Forensic South Community Service Balint group n = 5. For the evaluation period the job roles were solely clinical nurse specialists, taking a snapshot of the group between September 2020 and January 2022. The group met monthly for one hour virtually, led by Dr Arends, a specialist registrar in psychiatry with appropriate training in Balint leadership. The format in sessions was in keeping with the Balint method, as per The Balint Society, emphasising confidentiality. Data were gathered via survey tool, adapted from the literature using Likert scales and white space questions to identify barriers and facilitators.

**Results:**

Participants scored the group highly across the board in terms of acceptability, clinical impact, and fidelity measures. Notably 60% strongly agreed and 40% agreed the group was a safe place to express and process anxieties and frustrations about their work. All participants either agreed or strongly agreed the group had changed the way they think and practice, and that they felt able to consider their clinical encounters in a new light.

**Conclusion:**

Facilitators identified were of increased team working through cohesion and notably of increased appreciation for the functional and symbolic elements of the symptoms their patients presented with, suggesting that the value of the group existed in its providing of space to metabolise the often intense demands of Forensic patients, together and as a team. The main theme within barriers to the group processes were external in terms of other clinical demands requiring prioritisation.

